# Isolated diaphragmatic hydatid cyst: a rare entity in the paediatric population

**DOI:** 10.1093/jscr/rjae488

**Published:** 2024-08-10

**Authors:** Salma Mani, Habib Ullah Joya, Amani N Alansari, Sabrine Ben Youssef, Amine Ksia, Raed M Al-Zoubi

**Affiliations:** Pediatric Surgery Department, Fattouma Bourguiba University Hospital, Monastir 5000, Tunisia; University of Monastir, Monastir 5000, Tunisia; Department of Pediatric Surgery, Hamad Medical Cooperation, Doha 2001, Qatar; Department of Pediatric Surgery, Hamad Medical Cooperation, Doha 2001, Qatar; Pediatric Surgery Department, Fattouma Bourguiba University Hospital, Monastir 5000, Tunisia; University of Monastir, Monastir 5000, Tunisia; Pediatric Surgery Department, Fattouma Bourguiba University Hospital, Monastir 5000, Tunisia; University of Monastir, Monastir 5000, Tunisia; Surgical Research Section, Department of Surgery, Hamad Medical Corporation, Doha 2001, Qatar; Department of Biomedical Sciences, QU-Health, College of Health Sciences, Qatar University, Doha 2713, Qatar

**Keywords:** hydatid cyst, diaphragm, pediatric, 12 years old, abdominal pain

## Abstract

Isolated primary diaphragmatic hydatid disease (HD) occurs in approximately 1% of adult cases. However, this unique presentation of a pediatric diaphragmatic cystic mass has not been previously described in the literature. This study reports a rare case of a 12-year-old girl who was diagnosed with a diaphragmatic hydatid cyst. Surgical exploration via subcostal incision revealed an isolated cyst, free from the thoracic and abdominal viscera. Cystotomy, removal of daughter cysts, and excision of the pericyst cavity were performed, followed by diaphragmatic repair. Histopathological examination confirmed the diagnosis. The postoperative course was uneventful, and the patient completed an 8-week mebendazole regimen with no recurrence at 3 months’ follow-up. This paper recommends including HD in the differential diagnosis for pediatric patients presenting with diaphragmatic lesions, particularly in regions endemic for echinococcosis.

## Introduction

Hydatid disease (HD) is a parasitic disease caused by *Echinococcus granulosus*. It is a well-recognized zoonotic infection with worldwide endemicity, particularly in Europe, North and East Africa, the Middle East, Central Asia, Central and South America, and Australia. The liver (70%) and lungs (15–47%) are the most frequently affected organs [1], followed by the kidneys (2–4%). While less common, HD can involve other sites such as the brain, mediastinum, heart, bones, and even the kidneys. While it remains unreported amongst children [2], diaphragmatic HD is well documented in adults with an incidence of around 1% [3]. For instance, For instance, Eren *et al.* reported a rare case of a hydatid cyst in the diaphragm of a 34-year-old woman. The patient experienced right thoracoabdominal pain, and imaging revealed a giant lung cyst that extended into the thorax and abdomen. During surgery, an independent diaphragmatic cyst was found and removed along with over 200 daughter vesicles. The diaphragm was then repaired [3]. Additionally, Di Carlo *et al.* also reported a similar case in a 50-year-old woman who was initially diagnosed with a small calcified cystic mass in liver segment VII. However, surgery revealed that the cyst was attached to the diaphragm [4].

In the pediatric population, diaphragmatic hydatid cyst (DHC) is an extremely rare presentation. Some cases describe thoracic extension and respiratory complications [2]. Additionally, unusual abdominal locations like the retrovesical space and mesentery have been documented [5, 6]. To the best of our knowledge, an isolated type of HDC has yet to be reported. Herein, this study presents a unique and the first case of a pediatric patient with an isolated hydatid cyst confined to the diaphragm, without involvement of the thoracic or abdominal viscera.

## Case report

A 12-year-old girl was referred to the emergency department with vague acute right upper abdominal pain radiating to the right shoulder for a 3-day duration. She experienced no fever or dyspnea. A review of other systems revealed no significant findings. Vital signs on presentation were temperature 37°C, heart rate 98 bpm, respiratory rate 18 rpm, and blood pressure 112/71 mmHg. Physical examination proved mild tenderness to palpation in the right hypochondrium, but the remainder of the examination, including the respiratory system, was normal.

**Figure 1:**
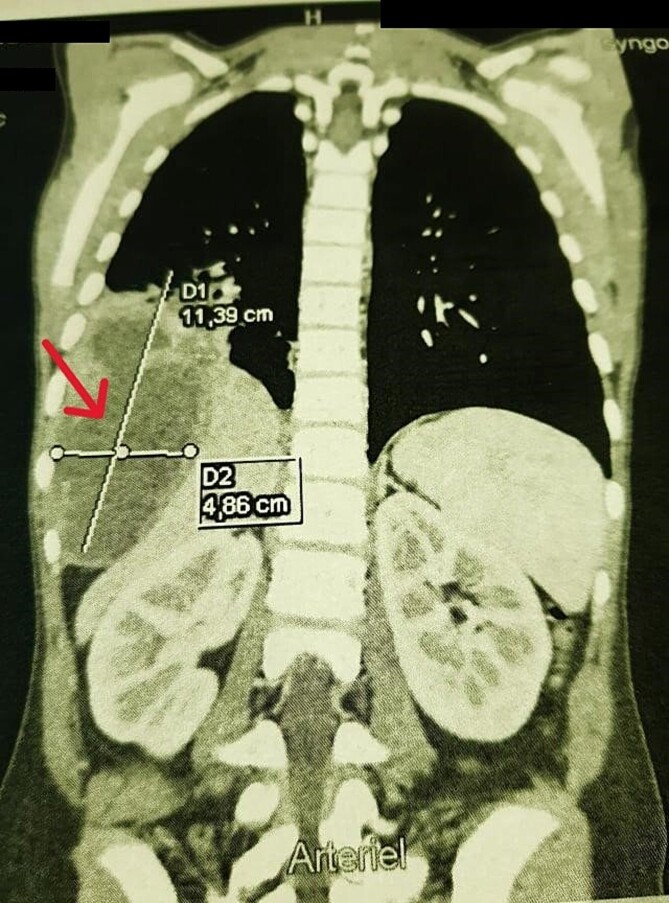
Coronal chest CT view 11 × 4 cm^2^ hydatid cyst that seems arising from the liver with possible intrathoracic rupture.

**Figure 2:**
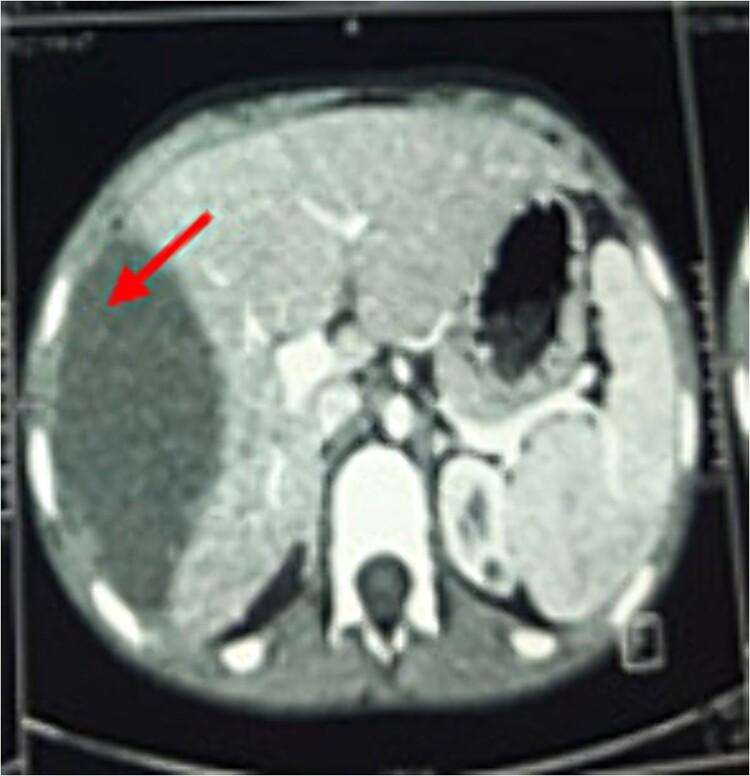
Axial chest CT view of the same hydatid cyst.

A chest X-ray demonstrated right hemi-diaphragmatic elevation. Given this finding, a computed tomography scan (CT) of the chest and abdomen was performed for further evaluation. The CT report concluded the presence of a hydatid cyst originating from the hepatic dome with a suspected intrathoracic extension (Figs 1 and 2). Serological testing for echinococcosis was positive.

**Figure 3:**
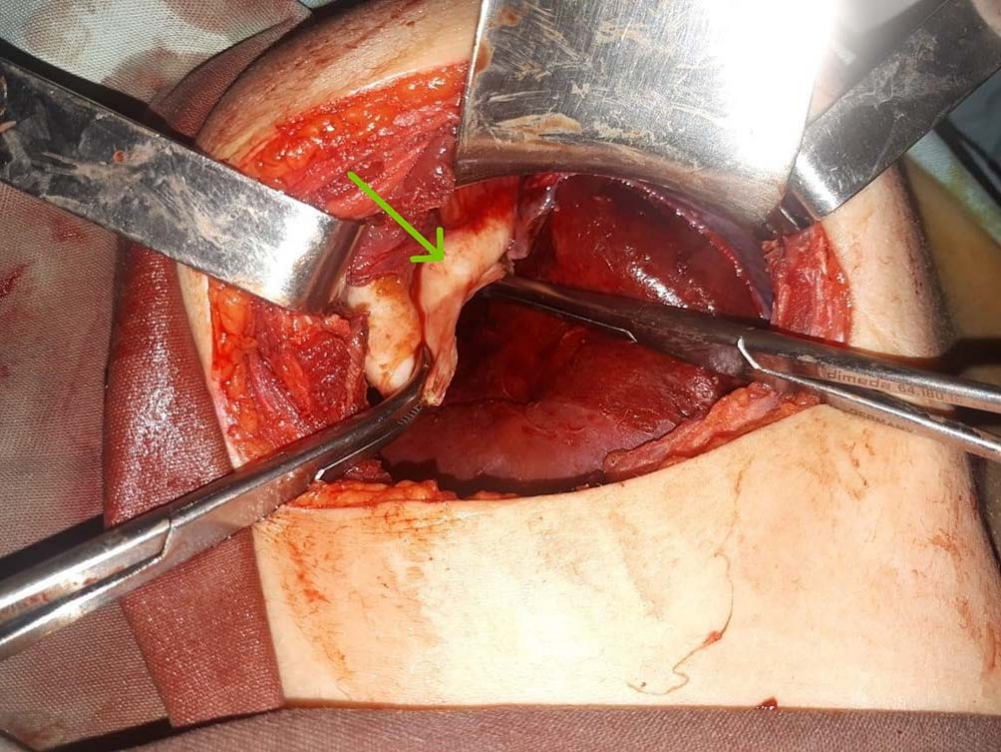
Intraoperative findings. (a) Location of the DHC during resection without adherence to the lung or abdominal viscera. (b) Germinative membrane of the DHC.

A laparotomy via a right subcostal incision was performed. Exploration identified a unilocular, non-adherent hydatid cyst localized solely to the diaphragm, without adherence to the lung or abdominal viscera (Fig. 3a and b). A cystotomy was performed with the removal of multiple daughter vesicles. The pericyst cavity was excised, and the diaphragmatic defect was repaired. Intraoperative inspection confirmed diaphragmatic integrity and no lung involvement. A histopathological examination confirmed the diagnosis of HD. Postoperatively, the patient received oral mebendazole for 8 weeks, commencing on the first postoperative day. Three months of follow-up showed no clinical or radiological signs of recurrence.

## Discussion

Isolated DHCs pose a substantial diagnostic challenge. Hydatid serology often exhibits low sensitivity in these cases, potentially remaining negative even after surgery [7]. While chest radiography, ultrasonography, and CTs can be valuable diagnostic tools [3], these modalities may not definitively identify DHCs. In our case, the CT scan misdiagnosed the lesion as a ruptured hepatic hydatid cyst with suspected thoracic extension. Although CT excels at visualizing the location and extent of hydatid cysts in commonly affected organs, it may struggle to depict the diaphragmatic entanglement clearly [8]. Magnetic resonance imaging (MRI) offers superior delineation of diaphragmatic pathologies and could potentially have facilitated a more accurate preoperative localization in our case [8]. Definitive diagnosis of DHCs hinges on the histological identification of muscle fibres within the pericyst [9]. Despite these advancements, the majority of reported DHCs were preoperatively misdiagnosed as liver or lung hydatid cysts, with the true location only becoming apparent intraoperatively [1]. Our case was not an exception. A ruptured hepatic hydatid cyst was suspected preoperatively.

Surgery is the cornerstone of DHC treatment. The chosen surgical approach (thoracotomy, midline laparotomy, or subcostal laparotomy) depends on the cyst’s location within the diaphragm [7]. Notably, good long-term outcomes have been achieved with simple removal of the cyst membrane, without complete pericyst excision [1, 9]. Diaphragmatic repair is typically performed after cyst removal to prevent potential herniation [1, 3, 9, 10].

## Conclusion

Although isolated primary DHCs are rare in children, they warrant inclusion in the differential diagnosis for pediatric patients presenting with diaphragmatic lesions, particularly in endemic regions like the Middle East and Europe. Preoperative diagnosis remains challenging, and MRI should be considered, when available, for precise cyst localization and anatomical relations. Surgical intervention with complete cyst excision and meticulous diaphragmatic repair represents the gold standard of treatment.

## Data Availability

Data will be made available on request.
